# Supplementary material for: Optogenetic strategies for high-efficiency all-optical interrogation using blue-light-sensitive opsins
Source: eLife. 2021 May 25;10:e63359. doi: 10.7554/eLife.63359 (PMC8177884; doi:10.7554/eLife.63359)
Supplement: Supplementary file 1. — Supplementary Table 1.Photocurrents elicited by two-photon spiral scan over the soma for CoChR variants. ‘v’ marks under each post hoc p value denote which CoChR variants were compared post hoc to obtain each specific p value. This also applies to Supplementary Table 2–4. # indicates that a separate Kruskal-Wallis test with separate post hoc comparisons was performed for comparing soCoChR under 40 mW with stCoChR and CoChR, since the two light powers used for soCoChR are paired. The row of soCoChR @ 40 mW presents the results of this separate set of tests, in which soCoChR @ 40 mW replaces soCoChR in the post hoc comparisons. This applies also to Supplementary Table 3–4. Supplementary Table 2. Photocurrents elicited by one-photon full-field illumination of CoChR variants. Supplementary Table 3. Soma-to-full-field photocurrent ratio of CoChR variants. Supplementary Table 4. τdecay of photocurrent with distance from soma along neurites for CoChR variants. [file elife-63359-supp1.docx]

|  |  |  |  | Kruskal-Wallis test | | p values for post hoc comparisons | | |
| --- | --- | --- | --- | --- | --- | --- | --- | --- |
| Variant | n cells | Mean (pA) | SEM (pA) | *χ*^2^ | p value | 9.2E-3 | 3.8E–6 | 0.11 |
| Non-targeted CoChR | 11 | 712.3 | 188.9 | 23.8 | 6.9E–6 | v |  | v |
| stCoChR | 10 | 1927.4 | 283.3 |  |  |  | v | v |
| soCoChR | 10 | 14.9 | 4.2 |  |  | v | v |  |
| soCoChR @ 40 mW ^#^ | 9 | 22.6 | 5.4 | 22.6 | 1.2E–5 | 0.01 | 6.2E–6 | 0.09 |

**Supplementary table 1. Photocurrents elicited by two-photon spiral scan over the soma for CoChR variants.** “v” marks under each post hoc p value denote which CoChR variants were compared post hoc to obtain each specific p value. This also applies to Table S2–S4. ^#^ indicates that a separate Kruskal-Wallis test with separate post hoc comparisons was performed for comparing soCoChR under 40 mW with stCoChR and CoChR, since the two light powers used for soCoChR are paired. The row of soCoChR @ 40 mW presents the results of this separate set of tests, in which soCoChR @ 40 mW replaces soCoChR in the post hoc comparisons. This applies also to Table S3–S4.

|  |  |  |  | Kruskal-Wallis test | | p values for post hoc comparisons | | |
| --- | --- | --- | --- | --- | --- | --- | --- | --- |
| Variant | n cells | Mean (pA) | SEM (pA) | *χ*^2^ | p value | 2E-4 | 2.2E–5 | 0.87 |
| Non-targeted CoChR | 14 | 3296.3 | 452.4 | 23.3 | 8.5E–6 | v |  | v |
| stCoChR | 14 | 3709.5 | 381.2 |  |  |  | v | v |
| soCoChR | 11 | 91.8 | 9.7 |  |  | v | v |  |

**Supplementary table 2. Photocurrents elicited by one-photon full-field illumination of CoChR variants.**

|  |  |  |  | Kruskal-Wallis test | | p values for post hoc comparisons | | |
| --- | --- | --- | --- | --- | --- | --- | --- | --- |
| Variant | n cells | Mean | SEM | *χ*^2^ | p value | 0.80 | 1.3E–3 | 8.4E-3 |
| Non-targeted CoChR | 11 | 0.18 | 0.04 | 14.2 | 8.2E–4 | v |  | v |
| stCoChR | 10 | 0.53 | 0.06 |  |  |  | v | v |
| soCoChR | 10 | 0.15 | 0.03 |  |  | v | v |  |
| soCoChR @ 40 mW ^#^ | 9 | 0.22 | 0.04 | 13.4 | 1.2E-3 | 0.80 | 1.9E-2 | 1.4E-3 |

**Supplementary table 3. Soma-to-full-field photocurrent ratio of CoChR variants.**

|  |  |  |  | Kruskal-Wallis test | | p values for post hoc comparisons | | |
| --- | --- | --- | --- | --- | --- | --- | --- | --- |
| Variant | n cells | Mean (µm) | SEM (µm) | *χ*^2^ | p value | 0.43 | 0.46 | 3.8E-2 |
| Non-targeted CoChR | 11 | 35.4 | 6.6 | 6.0 | 4.97E-2 | v |  | v |
| stCoChR | 10 | 16.1 | 1.9 |  |  |  | v | v |
| soCoChR | 10 | 27.2 | 6.2 |  |  | v | v |  |
| soCoChR @ 40 mW ^#^ | 9 | 17.2 | 3.2 | 8.6 | 1.4E-2 | 2.3E-2 | 0.94 | 4.9E-2 |

**Supplementary table 4. τ_decay_ of photocurrent with distance from soma along neurites for CoChR variants.**
